# Role of Ultrasonography in the Diagnosis of Splenic Diseases

**DOI:** 10.3390/diagnostics16203294

**Published:** 2026-10-10

**Authors:** Hiroko Naganuma, Hideaki Ishida

**Affiliations:** 1 Department of Gastroenterology, Yokote Municipal Hospital, Akita 013-8602, Japan; 2 Department of Gastroenterology, Akita Red Cross Hospital, Akita 010-1495, Japan

**Keywords:** wide scanning, contrast-enhanced ultrasound, microvascular flow imaging, ultrasound elastography, spleen

## Abstract

The spleen, which serves as an important indicator of disease activity in various disorders, is routinely examined during abdominal ultrasonography. Although splenic ultrasonography has received relatively little attention compared with that focused on the liver and pancreas, recent advances in ultrasound technology have markedly improved its diagnostic capability. This mini-review re-evaluates the current role of ultrasonography in splenic imaging, focusing on recent technological developments and their clinical applications. Wide-scanning (WS) technology expands the field of view from approximately 70° to 140°, enabling visualization of the entire spleen within a single image. This may facilitate more reliable assessment of splenic size and peripheral lesions and can be combined with Doppler ultrasonography and contrast-enhanced ultrasound (CEUS). Microvascular flow imaging (MVFI) techniques, including Superb Microvascular Imaging and B-flow imaging, provide high-frame-rate imaging with excellent sensitivity for slow blood flow, allowing more detailed assessment of the intralesional vascularity and potentially aiding the evaluation of splenic tumors and infarctions. Splenic elastography offers a non-invasive assessment of portal hypertension and, when interpreted together with liver stiffness, may provide additional diagnostic information in non-cirrhotic portal hypertension. CEUS provides real-time assessment of splenic perfusion and lesion enhancement and may aid the characterization of focal lesions and the evaluation of splenic infarction. However, spleen-specific evidence remains limited, with much of the available literature consisting of small observational studies, case series, and case reports. Based on the available literature and our clinical experience, we also propose a practical multiparametric approach to splenic ultrasonography; however, this approach should be regarded as a clinically oriented framework rather than a prospectively validated diagnostic algorithm.

## 1. Introduction

**Figure 1:**
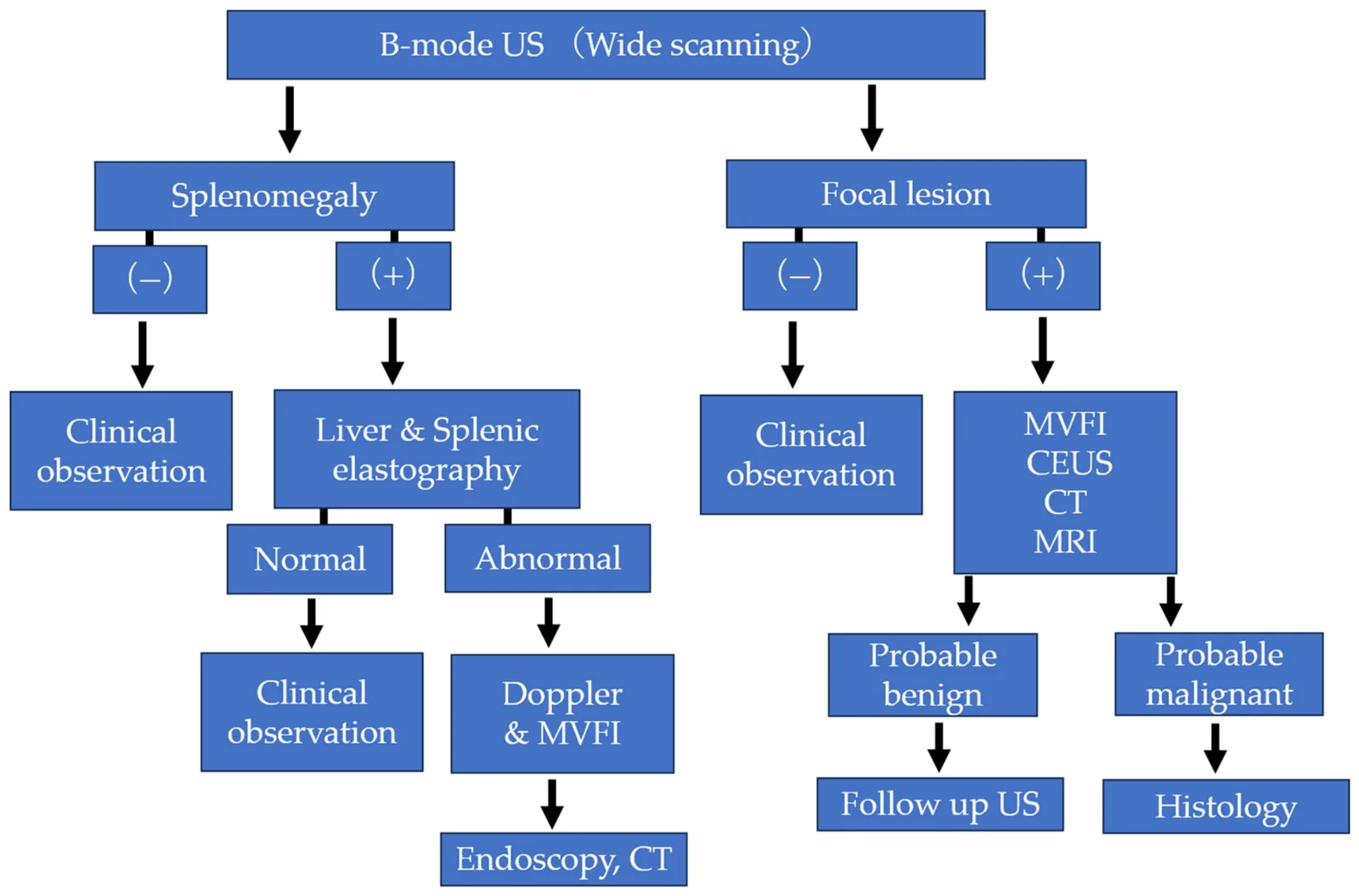
Recommended diagnostic approach to splenic ultrasonography in clinical practice. US, ultrasound; MVFI, microvascular flow imaging; CEUS, contrast-enhanced ultrasound; CT, computed tomography; MRI, magnetic resonance imaging.

Although the spleen should always be evaluated during routine abdominal ultrasonography (US), its sonographic assessment has not been extensively discussed in the literature [1,2,3,4,5,6]. In many cases, ultrasonography is performed alongside CT and MRI without an in-depth evaluation of its findings; in other words, the diverse inherent benefits of US are frequently underutilized. Meanwhile, recent remarkable technological advances in US have enabled clinicians to re-examine the abdominal organs from multiple perspectives [7,8,9]. In this narrative review, a literature search was conducted using PubMed for relevant articles published between January 2000 and September 2026. The search terms included “spleen”, “ultrasound”, “ultrasonography”, “contrast-enhanced ultrasound”, “microvascular imaging”, “superb microvascular imaging”, “B-flow”, “hand-held ultrasound”, “wide scanning”, and “wide view”. Additional relevant publications were identified by manually reviewing the reference lists of selected articles. The general inclusion criteria were as follows: (1) the study provided extractable clinical information, including relevant medical imaging findings; (2) the study provided clinically relevant information regarding the application, diagnostic performance, technical characteristics, or limitations of ultrasonographic techniques, or relevant technical information on the ultrasound techniques discussed in this review. Conference abstracts and posters were not included because the available information was insufficient for adequate evaluation of study quality and methodology. Recent publications were preferred, but earlier key studies were included when considered important for understanding the development or clinical application of the techniques discussed. Although this review focuses primarily on recent advances in splenic ultrasonography, conventional techniques and earlier key studies are also discussed where necessary to provide clinical and technical context for newer applications. Based on the available evidence and our clinical experience, we propose the following approach. When splenomegaly is identified by B-mode ultrasonography or WS in patients with suspected liver or portal venous disease, spleen stiffness measurement may provide additional information regarding the presence and severity of portal hypertension. When portal hypertension is suspected, Doppler ultrasonography and, where available, MVFI may be useful for further assessment of portal hemodynamics, collateral vessels, and portal vein thrombosis. Endoscopy or CT should subsequently be considered when clinically indicated. Our recommended diagnostic pathway for US examination of splenomegaly and focal splenic lesions is summarized in Figure 1. This pathway represents a clinically oriented framework and has not been prospectively validated as a diagnostic algorithm.

## 2. Wide Scanning (WS)

**Figure 2:**
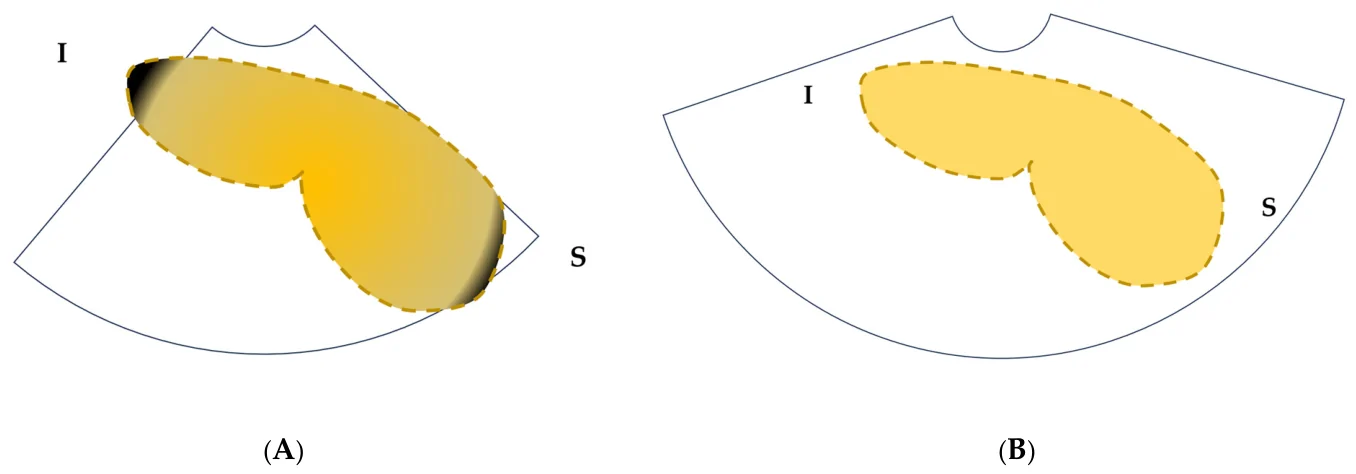
Schematic illustration of wide scanning. (**A**) Conventional scanning; (**B**) wide scanning. Wide scanning provides a field of view of approximately 140° (maximum field of view with the Canon Aplio i800 system), which is substantially wider than that of conventional scanning (approximately 70°). I, inferior; S, superior. The splenic images are intentionally displayed in an orientation that facilitates continuous anatomical visualization from the pancreatic tail through the splenic hilum to the splenic parenchyma.

WS is a field-of-view (FOV) expansion technique that controls the activation timing of individual transducer elements to steer ultrasound beams laterally (Figure 2). In the cited study using the Aplio i800 system (Canon medical systems corporation, Kawasaki, Japan), this beam-steering technology expanded the scanning angle from approximately 70° to 140° [10], allowing a substantially wider anatomical area to be visualized within a single image. This wider FOV is particularly advantageous for splenic imaging, as it facilitates visualization of the entire spleen, including its peripheral margins, which may be difficult to assess with conventional scanning.

In modern systems, WS can be activated simply by selecting the corresponding imaging mode. In addition to increasing the scanning angle, WS expands the lateral footprint at depth; in the same study described above, the footprint increased from approximately 240 mm to 310 mm [10]. This expanded footprint is clinically relevant for splenic imaging because it increases the likelihood of encompassing the entire spleen within a single image. WS can be used not only for B-mode imaging but also for Doppler studies and CEUS.

### 2.1. Clinical Utility of WS

**Figure 3:**
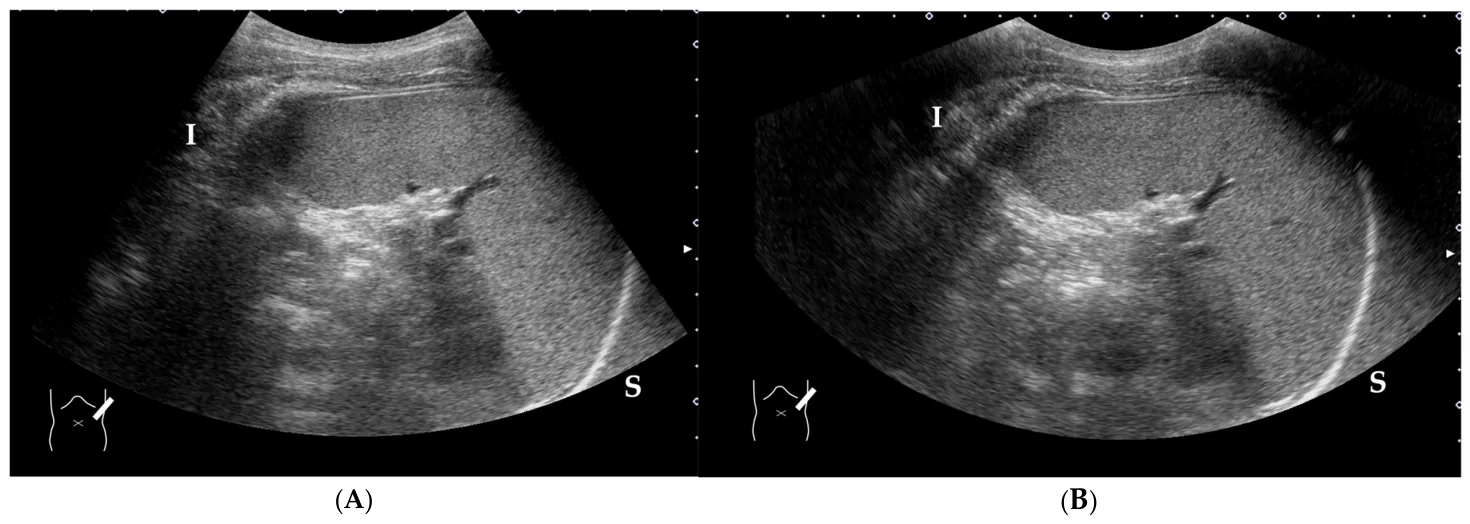
Splenic sonograms. (**A**) Conventional scanning; (**B**) wide scanning. Wide scanning provides improved visualization of the splenic margin compared with conventional scanning in this representative case. I, inferior; S, superior. The splenic images are intentionally displayed in an orientation that facilitates continuous anatomical visualization from the pancreatic tail through the splenic hilum to the splenic parenchyma.

One of the most common difficulties encountered when scanning the spleen is the insufficient ability to evaluate the splenic margins due to acoustic interference from the adjacent aerated lung. As a result, it is challenging to accurately measure the splenic size, and subtle marginal lesions may be overlooked. WS may facilitate visualization of the splenic margins and help overcome some of the limitations encountered with conventional scanning (Figure 3). A recent comparative study also demonstrated improved agreement of spleen size measurements with CT/MRI using wide-view compared with conventional ultrasonography [10]. Moreover, vessels around the spleen—including aneurysms, splenic vein thrombosis, and collateral pathways—can be easily evaluated without the need for delicate probe manipulations (e.g., extreme intercostal angulation or rotation).

### 2.2. Limitations of WS

As expected, WS slightly degrades image quality at the deepest and lateral-most regions, particularly in obese patients [9,10]. However, this aspect is anticipated to improve as interpolation algorithms are refined.

In routine clinical practice, if WS demonstrates splenomegaly (generally defined as a splenic longitudinal diameter of ≥13 cm) in asymptomatic individuals or patients with suspected liver disease, spleen stiffness measurement may provide additional information regarding the presence and severity of portal hypertension. Conversely, in patients with trauma or left upper quadrant pain, if WS reveals a focal area with altered parenchymal echotexture in the splenic periphery or perisplenic fluid collection, splenic infarction or traumatic splenic injury should be suspected. In such cases, MVFI may be useful for assessing blood flow within the lesion, followed by CEUS when further characterization is clinically required. This sequence may serve as a practical approach to guide further imaging assessment in daily clinical practice, although it has not been prospectively validated. Clinical evidence regarding WS remains limited because it is a relatively new technique, and further studies are needed to establish its clinical utility.

## 3. Microvascular Flow Imaging (MVFI)

**Figure 4:**
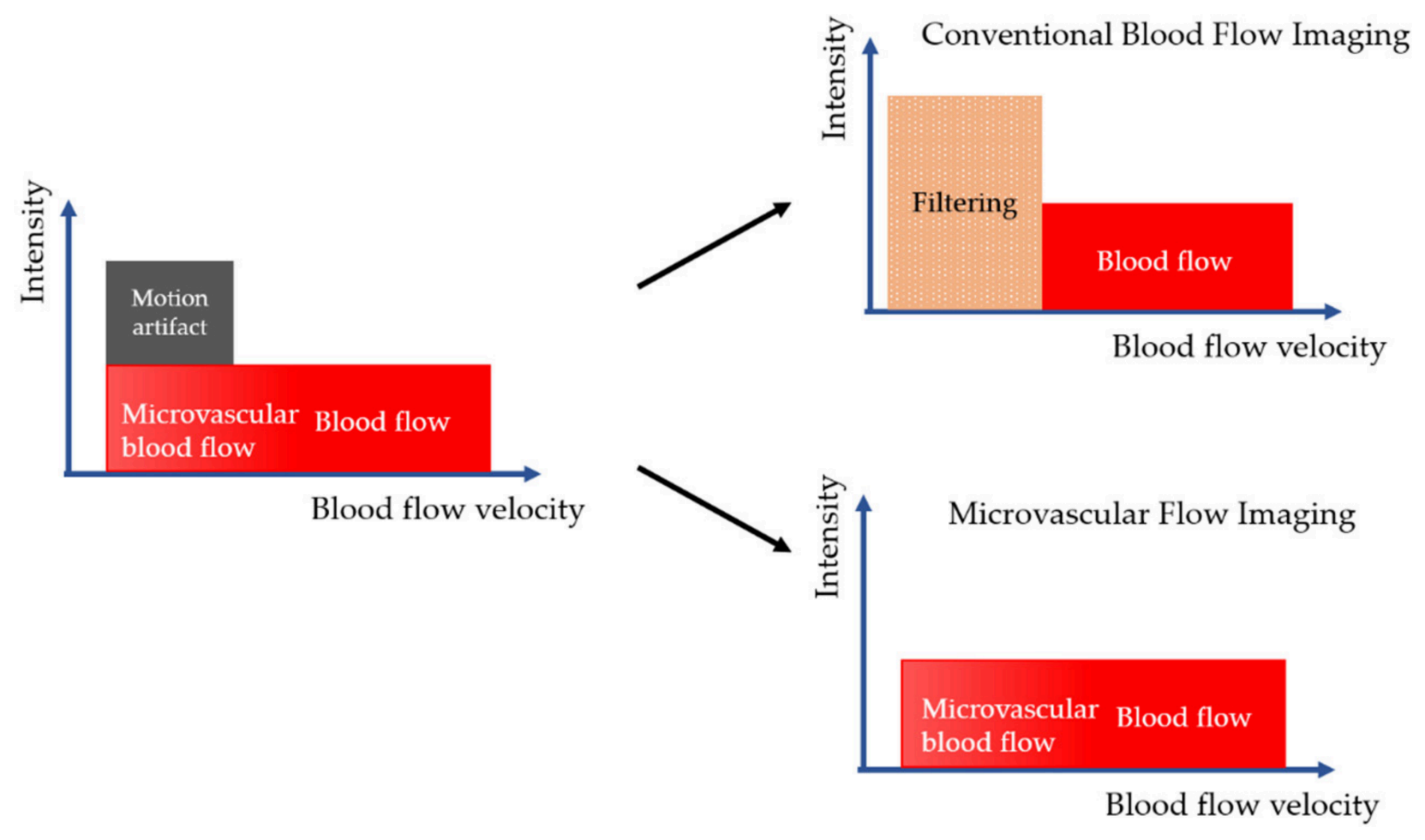
Schematic illustration of MVFI technology (1). MVFI selectively suppresses tissue-derived motion artifacts while preserving blood flow signals, thereby enabling highly sensitive visualization of low-velocity blood flow.

**Figure 5:**
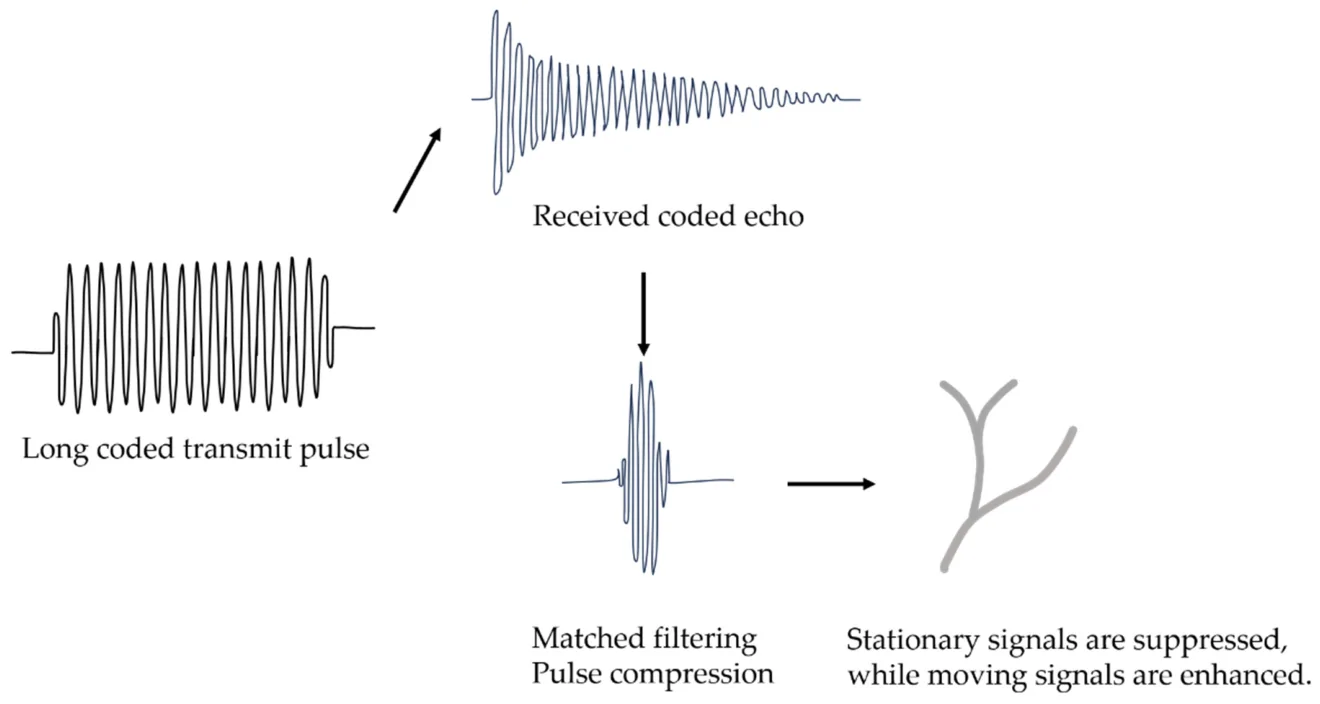
Schematic illustration of MVFI technology (2): B-flow imaging. B-flow is a type of digitally encoded ultrasound. Coding on transit with a broadband, temporally elongated pulse and subsequent decoding on receive pulse results in improved signal-to-noise ratio through increased peak acoustic power.

MVFI was developed to overcome the major limitation of conventional Doppler imaging: insufficient sensitivity for detecting slow flow. At present, the diagnostic technologies collectively referred to as MVFI mainly utilize two different techniques: B-flow imaging, a type of digitally coded imaging, and superb microvascular imaging (SMI), which uses spatio-temporal correlation processing (Figure 4 and Figure 5). MVFI can demonstrate altered, reduced, or low-velocity flow in cases where conventional Doppler assessment is inconclusive [8,9,11,12]; however, these findings do not by themselves establish the presence or cause of thrombosis. For hypovascular tumors, MVFI may improve visualization of vascular architecture in some lesions. In short, MVFI improves visualization of slow-velocity flow and may provide vascular information complementary to that obtained with CEUS. SMI is optimized for the visualization of low-velocity microvascular flow, with the velocity scale typically set below approximately 2.5 cm/s [13]. Despite its potential value, quantitative diagnostic performance data for splenic applications of MVFI, including sensitivity, specificity, positive predictive value (PPV), and negative predictive value (NPV), remain limited, and further prospective studies are required.

Although SMI and B-flow imaging are grouped under the practical umbrella term “MVFI” in this review, they are based on different proprietary technologies and should not be regarded as technically equivalent. Their imaging characteristics and performance are manufacturer- and system-dependent, and standardized acquisition parameters and diagnostic criteria have not yet been established.

### 3.1. Clinical Utility of MVFI

**Figure 6:**
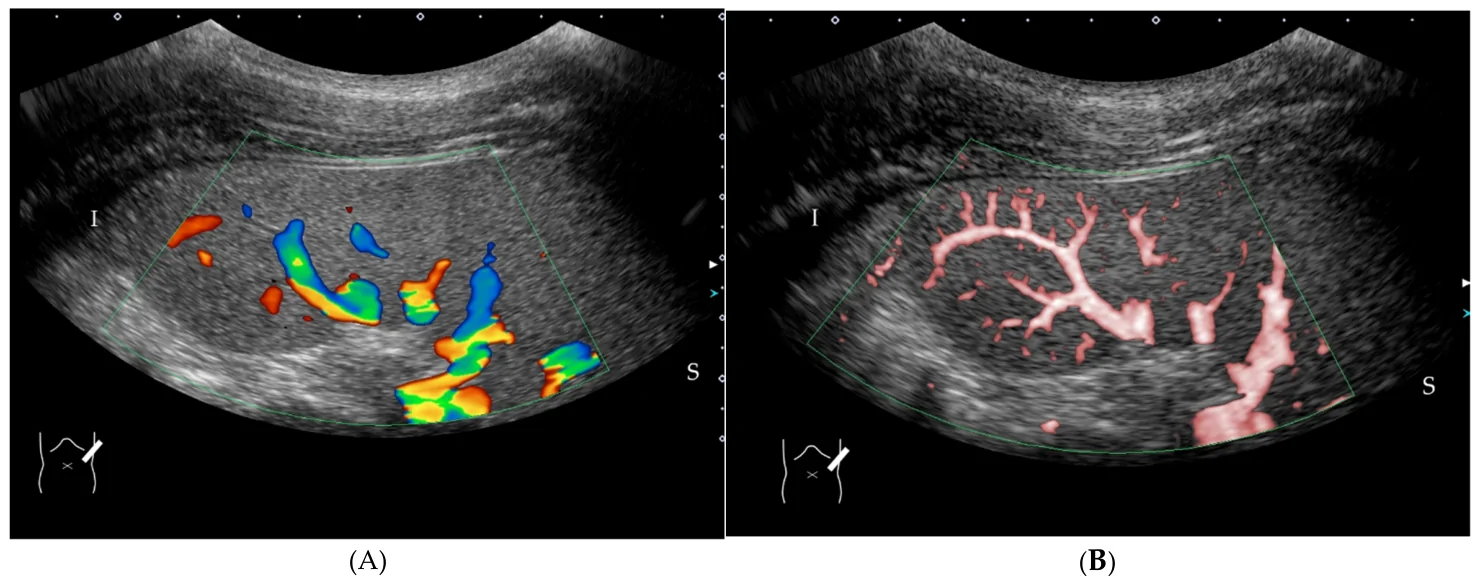
Color Doppler and MVFI image of a normal spleen. (**A**) Color Doppler imaging; (**B**) MVFI imaging. MVFI clearly demonstrates fine vascular structures within the normal splenic parenchyma. I, inferior; S, superior.

MVFI’s greatest advantage is its ability to display ultra-high-frame-rate images (approximately 50–80 frames/s), enabling real-time evaluation of dynamic flow changes. This may improve the characterization of subtle changes in blood flow (e.g., aneurysms or thrombosis) and the internal vasculature of tumors (Figure 6).

### 3.2. Limitations of MVFI

1. MVFI algorithms and processing methods differ among manufacturers and are largely proprietary. This lack of standardization may limit reproducibility and direct cross-platform comparison of imaging findings and diagnostic performance. Greater transparency and standardization of acquisition and processing methods would facilitate more robust multicenter evaluation of MVFI.
2. Because MVFI requires comparison of sequential high-frame-rate images and the observation field (ROI) is relatively restricted, wide-area evaluation is not feasible.
3. Near the heart or great vessels, motion-related artifacts may hinder the acquisition of adequate MVFI images.
4. As MVFI relies heavily on high-frequency components, visualization of deeper structures is sometimes insufficient.

Studies in non-splenic organs, including the breast and testis, have suggested that MVFI may provide vascular information complementary to that obtained with CEUS [13,14]. However, these findings represent indirect evidence and cannot be extrapolated to establish equivalent diagnostic performance of MVFI and CEUS for splenic lesions. At present, direct comparative evidence in splenic disease is limited, and further spleen-specific studies are required.

## 4. US Elastography

Ultrasound elastography comprises several techniques for estimating tissue stiffness; those currently used for spleen stiffness measurement are vibration-controlled transient elastography (VCTE), point shear-wave elastography (pSWE), and two-dimensional shear-wave elastography (2D-SWE) [15,16,17,18,19].

**Figure 7:**
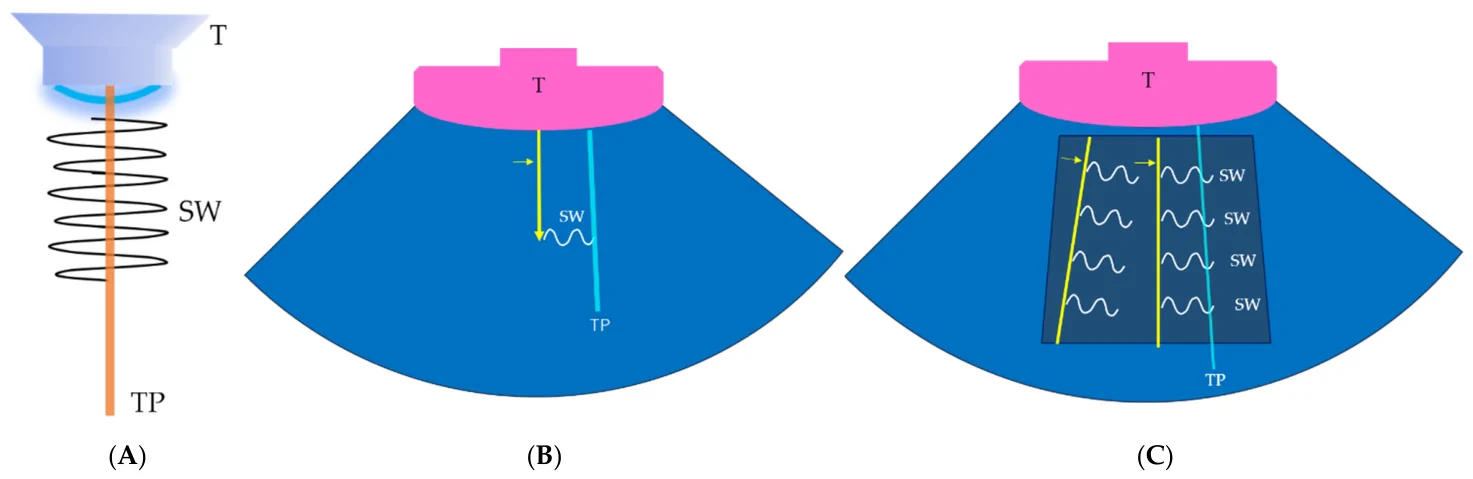
Schematic illustration of SW value measurement by ultrasound. (**A**) Conventional transient elastography. FibroScan probe contains a low-frequency mechanical vibration (50 Hz) and a single-element US transducer (5 MHz) with a focus at 35 mm. The propagation of a low-frequency elastic wave (shear wave) is measured by tracking US waves. A spleen-dedicated module uses a higher vibration frequency (100 Hz), as described in the text. (**B**) Point shear wave elastography. (**C**) 2D-shear wave elastography. T, transducer; SW, shear wave; TP, tracking pulse; arrow, push pulse.

**Figure 8:**
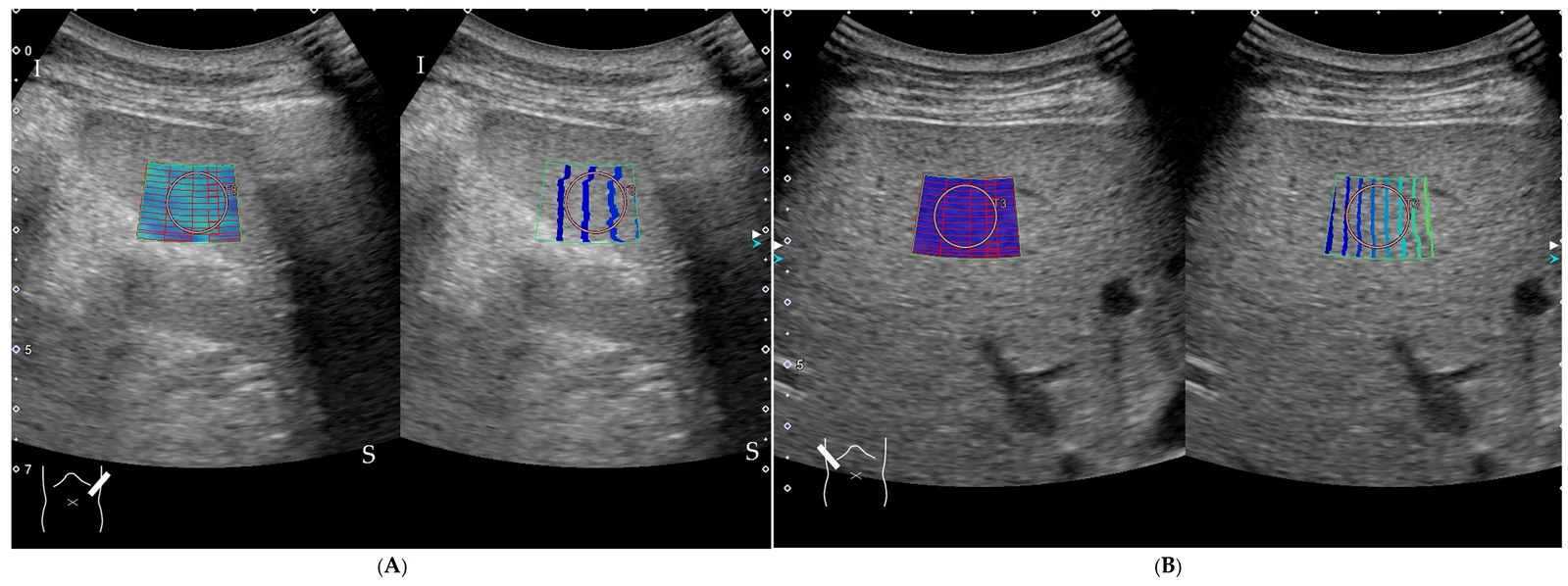
2DSWE images of a normal subject. US elastography shows a higher value in the spleen (**A**) (2.54 m/s) than in the liver (**B**) (1.37 m/s). I, inferior; S, superior.

**Figure 9:**
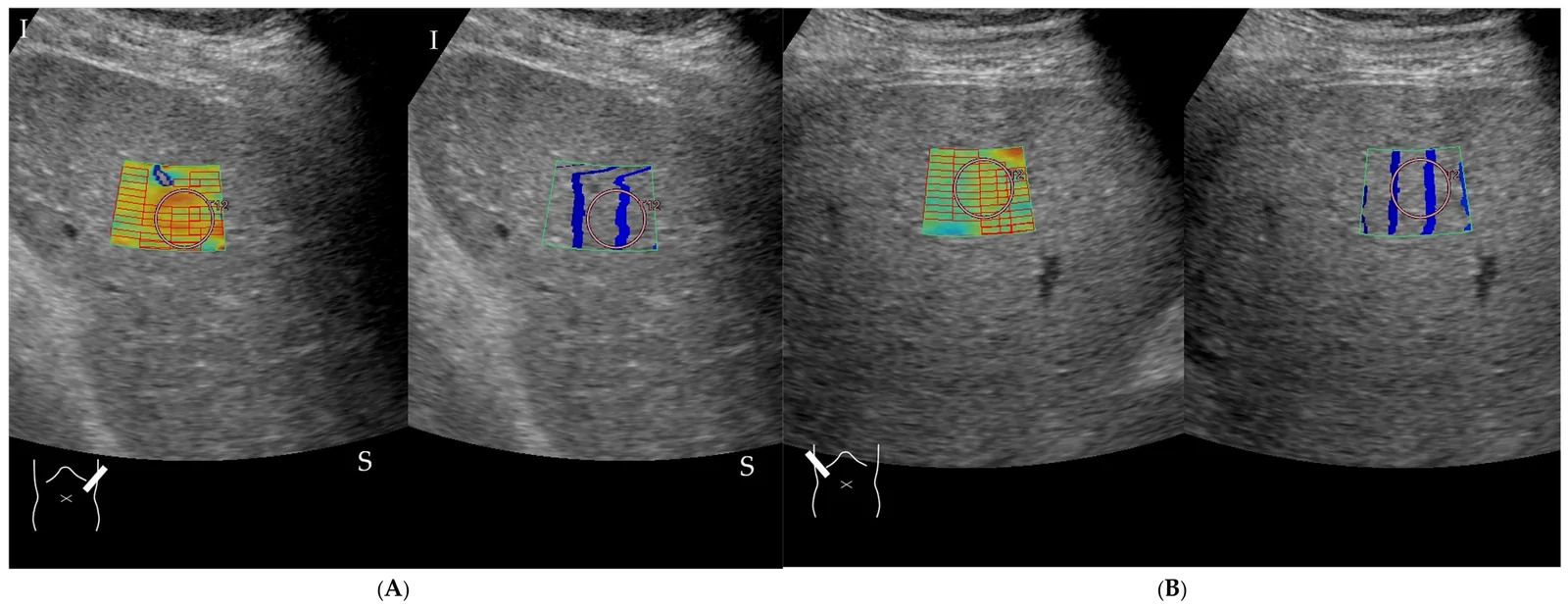
2DSWE images of an alcoholic liver cirrhosis. US elastography shows markedly higher values both in the spleen (**A**) (4.36 m/s) and in the liver (**B**) (3.63 m/s). I, inferior; S, superior.

In VCTE, a low-frequency mechanical vibration generated by the probe induces a shear wave, and its propagation is tracked ultrasonographically. A spleen-dedicated 100 Hz VCTE module was recently introduced to improve spleen stiffness measurement [16,18]. In pSWE, an acoustic radiation force impulse generates a localized shear wave within a small region of interest, and the shear-wave velocity is measured. In 2D-SWE, multiple acoustic radiation force impulses generate shear waves over a larger field, allowing two-dimensional stiffness mapping under B-mode guidance (Figure 7, Figure 8 and Figure 9) [15,20,21].

Importantly, these techniques differ in shear-wave generation, acquisition protocol, sampling area, operating frequency, and output format. Therefore, spleen stiffness values and diagnostic thresholds obtained using VCTE, pSWE, and 2D-SWE should not be regarded as directly interchangeable. Cut-off values should always be interpreted according to the specific elastographic technique and device used.

### 4.1. Clinical Utility of US Elastography

The major clinical value of splenic elastography is its ability to provide quantitative information related to portal hemodynamics that cannot be obtained from conventional B-mode imaging alone. Whenever possible, spleen stiffness should be interpreted together with liver stiffness and the underlying clinical context.

Measurement protocols differ among techniques. For example, recent studies using a spleen-dedicated 100 Hz VCTE module have emphasized the importance of repeated measurements to reduce intra-examination variability, whereas pSWE and 2D-SWE use different acquisition and quality-control criteria [16,18]. Accordingly, measurement number, region-of-interest placement, and quality criteria should follow technique- and device-specific recommendations rather than a single universal protocol.

### 4.2. Clinical Significance of US Elastography

The principal clinical application of spleen stiffness measurement is the non-invasive assessment of portal hypertension and the risk of gastroesophageal varices. However, reported thresholds vary substantially according to the elastographic technique used.

For spleen-dedicated VCTE, particularly the 100 Hz technique, values below approximately 21 kPa have been proposed to help rule out clinically significant portal hypertension, whereas values above approximately 50 kPa have been proposed to support its presence in appropriate clinical settings [15,22,23]. These thresholds are specific to VCTE-based measurements and should not be directly extrapolated to pSWE or 2D-SWE.

For pSWE/ARFI, stiffness is commonly expressed as shear-wave velocity (m/s). Furuichi et al. demonstrated that the relationship between spleen and liver stiffness, rather than an isolated spleen stiffness value, was useful for distinguishing idiopathic portal hypertension from cirrhosis [24].

For 2D-SWE, reported thresholds are generally lower than those obtained using spleen-dedicated VCTE and vary among systems. Using 2D-SWE, Zhou et al. reported substantially higher spleen stiffness values in patients with idiopathic portal hypertension than in patients with hepatitis B virus-related portal hypertension and demonstrated the usefulness of spleen stiffness for identifying high-risk varices [21].

Thus, the clinical interpretation of spleen stiffness should be technique-specific, and no single stiffness threshold can currently be applied universally across VCTE, pSWE, and 2D-SWE.

### 4.3. Limitations of US Elastography

1. Operators require training to obtain stable elastography measurements.
2. Measurement values vary across devices from different manufacturers.
3. Due to differing proprietary techniques, making cross-platform comparison difficult.
4. Transient elastography cannot be performed in patients with ascites.
5. The spleen is smaller in volume than the liver, so a non-negligible proportion of cases cannot be measured with this technique.

In routine clinical practice, elastography can provide useful complementary information in the initial assessment and follow-up of patients with liver disease and in patients with unusual varices. When clinically appropriate, paired assessment of liver and spleen stiffness may provide additional information regarding portal hemodynamics. When elastography suggests portal hypertension, it is reasonable to combine Doppler ultrasonography with MVFI to evaluate the presence of portosystemic collateral vessels and portal vein thrombosis.

## 5. Portable Ography in Splenic Assessment

### 5.1. Clinical Significance of Hand-Helportablenography

Portable and hand-held ultrasonography can be used as point-of-care ultrasound (POCUS) for rapid bedside assessment in various clinical settings. In addition to emergency and trauma settings, these devices can be used for bedside assessment of abdominal organs, including measurement of splenic size. Duminuco et al. demonstrated a high correlation between hand-held and conventional ultrasonography for spleen measurement in patients undergoing hematopoietic cell transplantation. Portable ultrasonography may also be useful in medically underserved or remote areas and in locations where installation or transport of conventional large ultrasound systems is difficult. Furthermore, the portability of these devices facilitates bedside examinations and may be useful for ultrasound education and training [25,26,27,28].

### 5.2. Limitations of Hand-Helportablenography

Compared with conventional high-end ultrasound systems, hand-held devices generally have a more limited range of advanced imaging applications and may provide lower image quality. Their diagnostic performance also depends on clinical application, device characteristics, and operator experience; therefore, appropriate ultrasound education and training are important to ensure effective clinical use [25,26,27,28].

## 6. Contrast-Enhanced Ultrasound (CEUS)

Contrast-enhanced ultrasound (CEUS) enables real-time assessment of the spatial distribution and temporal changes in microbubble contrast agents and is particularly useful for evaluating tissue perfusion and lesion vascularity [29,30]. Although CEUS is well established for hepatic lesion characterization, spleen-specific interpretation differs substantially because of the distinct histological and vascular architecture of the spleen [31]. Unlike hepatic CEUS, for which standardized diagnostic systems such as CEUS LI-RADS have been established, no comparable standardized classification system currently exists for splenic lesions.

In splenic CEUS, enhancement is generally assessed during an arterial phase, approximately 10–35 s after contrast injection, followed by a parenchymal phase. During the arterial phase, normal splenic parenchyma typically shows heterogeneous enhancement, known as the “zebra sign.” Consequently, lesion characterization relies substantially on enhancement behavior during the subsequent parenchymal phase. Malignant splenic lesions more frequently demonstrate washout, whereas benign lesions tend to show persistent enhancement or absent, mild, or delayed washout; however, these patterns represent general tendencies rather than definitive diagnostic criteria [32,33,34,35,36,37,38,39,40,41]. Ideally, CEUS characterization of splenic lesions should include the onset times of wash-in and washout, the degree of enhancement, and the temporal pattern of washout. However, quantitative data on these parameters remain limited for individual splenic diseases. Although terms such as “fast,” “slow,” and “incomplete” washout have been used in previous studies, standardized definitions of these patterns have not yet been established, making direct comparison among studies difficult. Nevertheless, evaluation of persistent enhancement or washout during the late phase, particularly at approximately 3–5 min after contrast administration, appears to provide useful information for differentiating splenic lesions. Further studies using standardized definitions and larger patient populations are needed to establish the diagnostic significance and performance of these CEUS findings.

Spleen-specific clinical applications of CEUS include characterization of focal splenic lesions, confirmation of splenic infarction, identification of accessory spleen or splenosis, and evaluation of splenic abnormalities in oncologic and post-traumatic settings [32,33,34,35,36,37,38,39,40,41].

### 6.1. Clinical Significance of CEUS

1. CEUS can be used to qualitatively characterize focal lesions and delineate the extent of ill-defined lesions.
2. Time–intensity curves (TICs) can be used for quantitative assessment.
3. This technique can detect abnormal perfusion areas, which are particularly useful for identifying splenic infarction.
4. Quantitative data on the diagnostic performance of CEUS, including sensitivity and specificity, remain limited for many individual splenic diseases because most available studies involve relatively small and heterogeneous patient populations.

### 6.2. Limitations of CEUS

This method is associated with a prolonged examination time. Image acquisition and interpretation are operator-dependent, and image quality may be affected by the acoustic window and imaging depth. The ultrasound contrast agents approved for clinical use and their approved indications vary among countries. In addition, differences in the characteristics and behavior of individual contrast agents should be considered when interpreting CEUS findings. Although ultrasound contrast agents generally have a favorable safety profile, potential adverse reactions should also be considered. CEUS also has a limited field of view, and quantitative assessment using time–intensity curves (TICs) may be affected by variability in image acquisition and measurement, potentially limiting repeatability.

In our proposed practical approach (Figure 1), when a focal splenic lesion is identified on B-mode imaging or when splenic infarction or trauma is suspected, MVFI may be used for further assessment of lesion vascularity and to identify an appropriate imaging plane for subsequent CEUS, when clinically indicated. This sequence represents a practical approach based on the available literature and our clinical experience and has not been prospectively validated as a diagnostic algorithm. When quantitative assessment using TIC is planned, image acquisition should be continued for a sufficient duration to capture the relevant enhancement and washout kinetics.

To facilitate comparison, the key features of MVFI and CEUS, including their technical characteristics and clinical applications, are summarized in Table 1.

**Figure 10:**
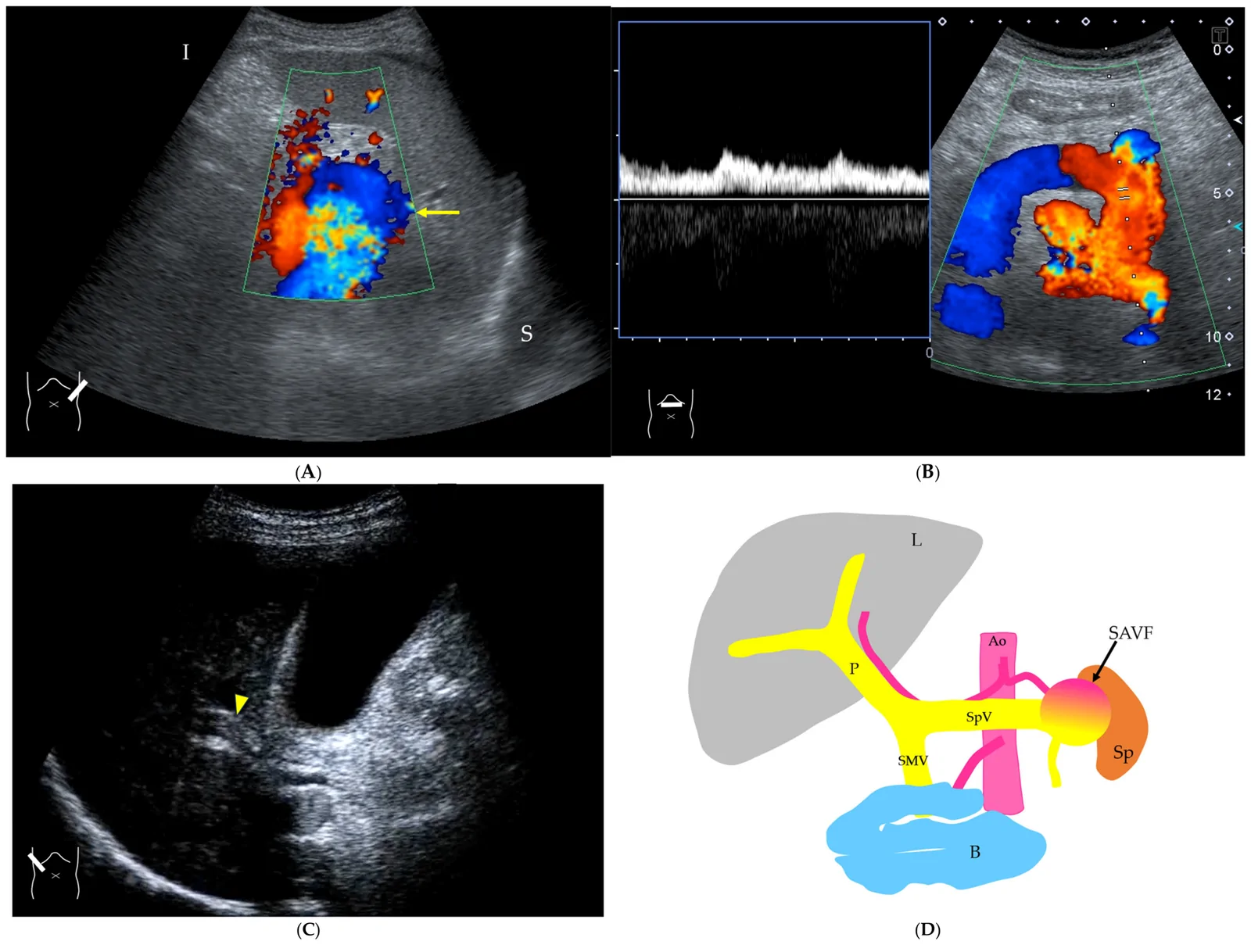
A splenic arteriovenous fistula. (**A**,**B**) Color Doppler sonography demonstrates turbulent flow within the splenic vein, raising suspicion of a splenic arteriovenous fistula (yellow arrow). (**C**) Contrast-enhanced sonogram of the intrahepatic portal vein. CEUS demonstrates rapid contrast enhancement of the portal vein (yellow arrow head), supporting the diagnosis of a splenic arteriovenous fistula. (**D**) Schematic drawing of the case. I, inferior; S, superior; SAVF, splenic arteriovenous fistula; L, liver; P, portal vein; SpV, splenic vein; SMV, superior mesenteric vein; Ao, aorta; Sp, spleen; B, edematous bowel. In this case, the combination of color Doppler ultrasonography and contrast-enhanced ultrasonography is effective in capturing the overall pathophysiology of the lesion.

**Figure 11:**
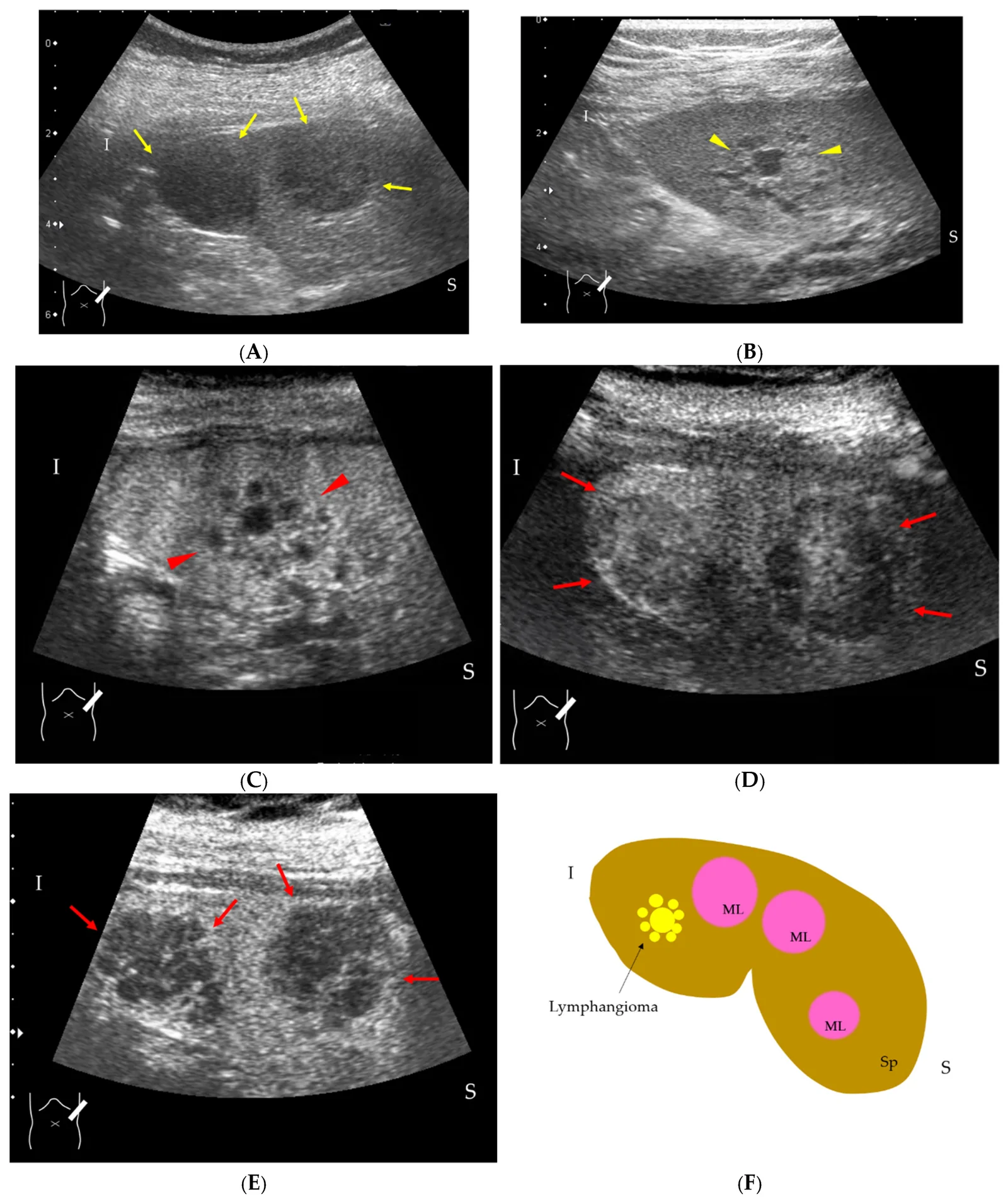
A case of simultaneous splenic lymphangioma and splenic lymphoma. (**A**,**B**) B-mode sonogram shows multiple focal lesions within the spleen (yellow arrows, lymphoma; yellow arrow heads, lymphangioma). (**C**,**D**) In the early phase of CEUS, the lymphangioma shows no enhancement (red arrowhead), whereas the lymphoma demonstrates faint enhancement (red arrow). (**E**) On CEUS, the lymphoma demonstrates washout in late phase (red arrows). Contrast-enhanced sonogram confirms the presence of two types of lesions with different enhancement characteristics. In this case, B-mode imaging combined with contrast-enhanced ultrasonography proved useful. (**F**) Schematic drawing of the case. ML, lymphoma; Sp, spleen; I, inferior; S, superior.

**Figure 12:**
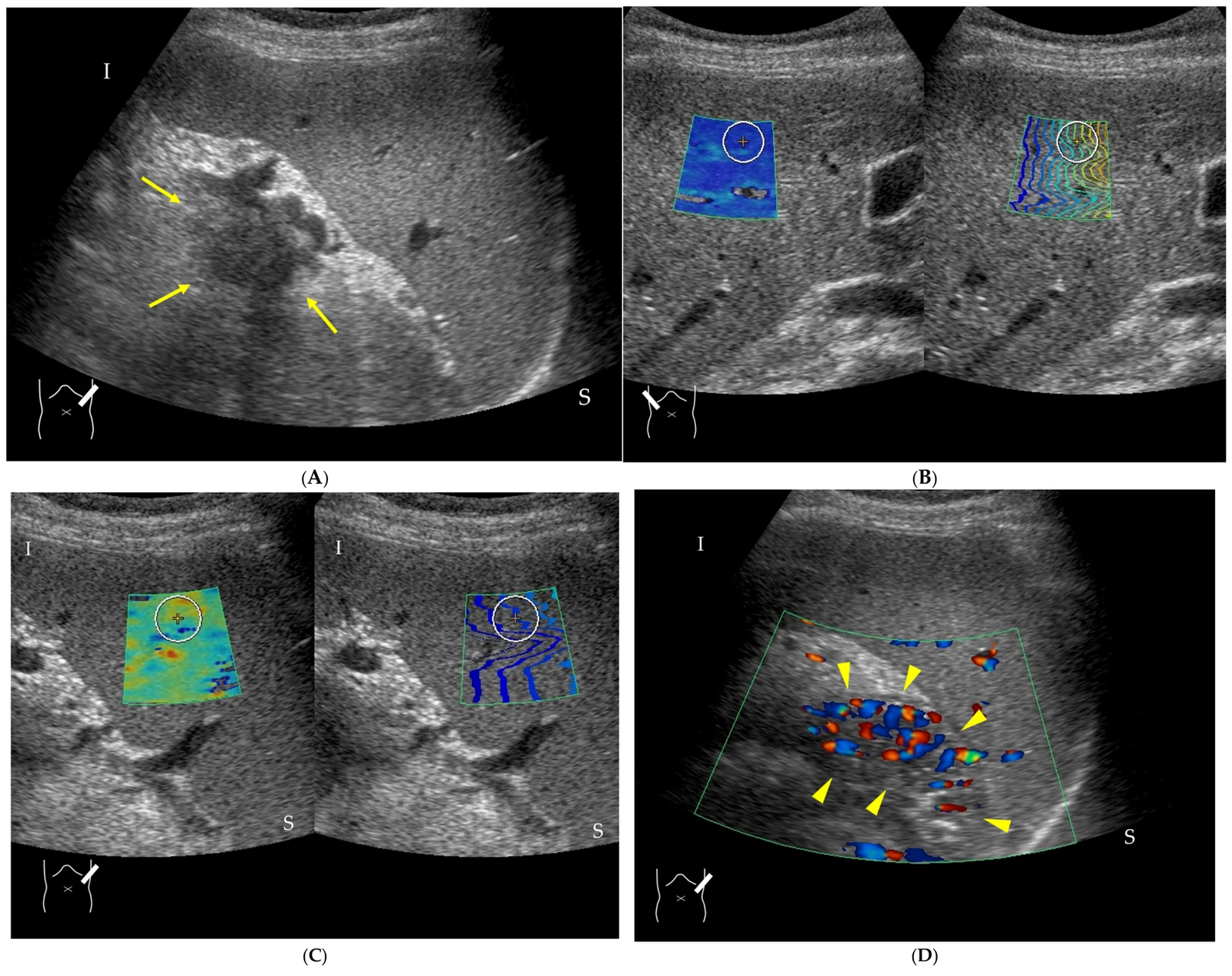
A case of left-sided portal hypertension due to pancreatic tail neuroendocrine tumor. (**A**) B-mode sonography reveals a 5 cm hypoechoic mass (yellow arrows) in the pancreatic tail; (**B**) liver SW value is 1.21 m/s, within the normal range; (**C**) the splenic SW value is 3.14 m/s, which is markedly higher than the normal range; (**D**) color Doppler sonogram reveals gastric varices (yellow arrowheads). In this case, US findings raise suspicion of left-sided portal hypertension. I, inferior; S, superior.

**Figure 13:**
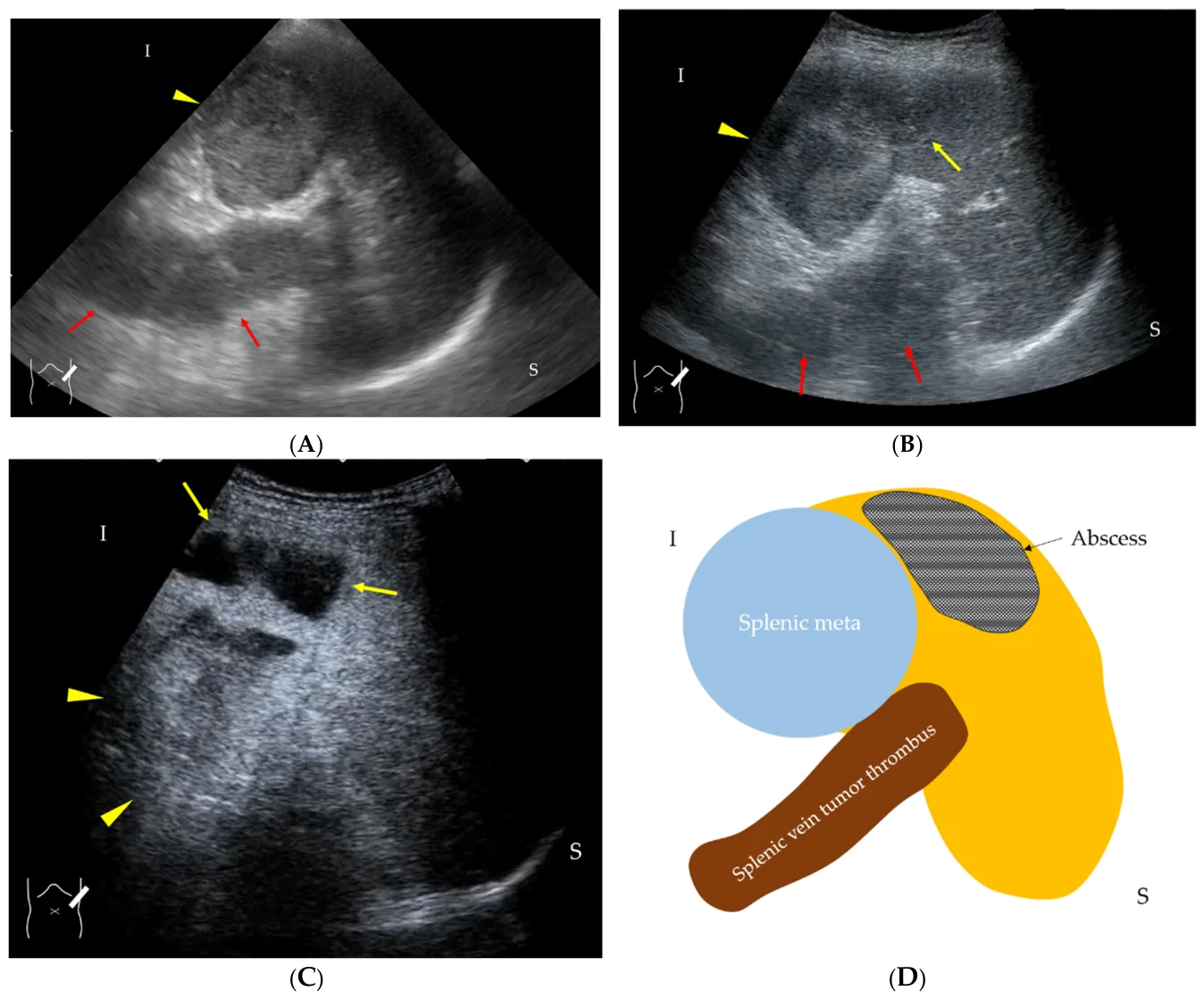
A splenic metastasis from gastric cancer complicated by splenic infarction. The patient presented with abdominal pain. (**A**) Handheld US demonstrates a splenic mass and a tumor thrombus in the splenic vein (yellow arrowhead, splenic metastasis; red arrows, splenic vein tumor thrombus). (**B**) High-end US reveals, in addition to the splenic metastasis and splenic vein tumor thrombus, an ill-defined hypoechoic area suspicious for splenic infarction (yellow arrowhead, splenic metastasis; red arrows, splenic vein tumor thrombus; yellow arrow, splenic infarction). (**C**) CEUS demonstrates the splenic metastasis as well as a non-enhancing area consistent with splenic infarction (yellow arrowhead, splenic metastasis; yellow arrows, splenic infarction). In this case, handheld US, high- end US, and CEUS each played complementary roles and contributed to the final diagnosis. (**D**) Schematic illustration of this case. I, inferior; S, superior.

**Figure 14:**
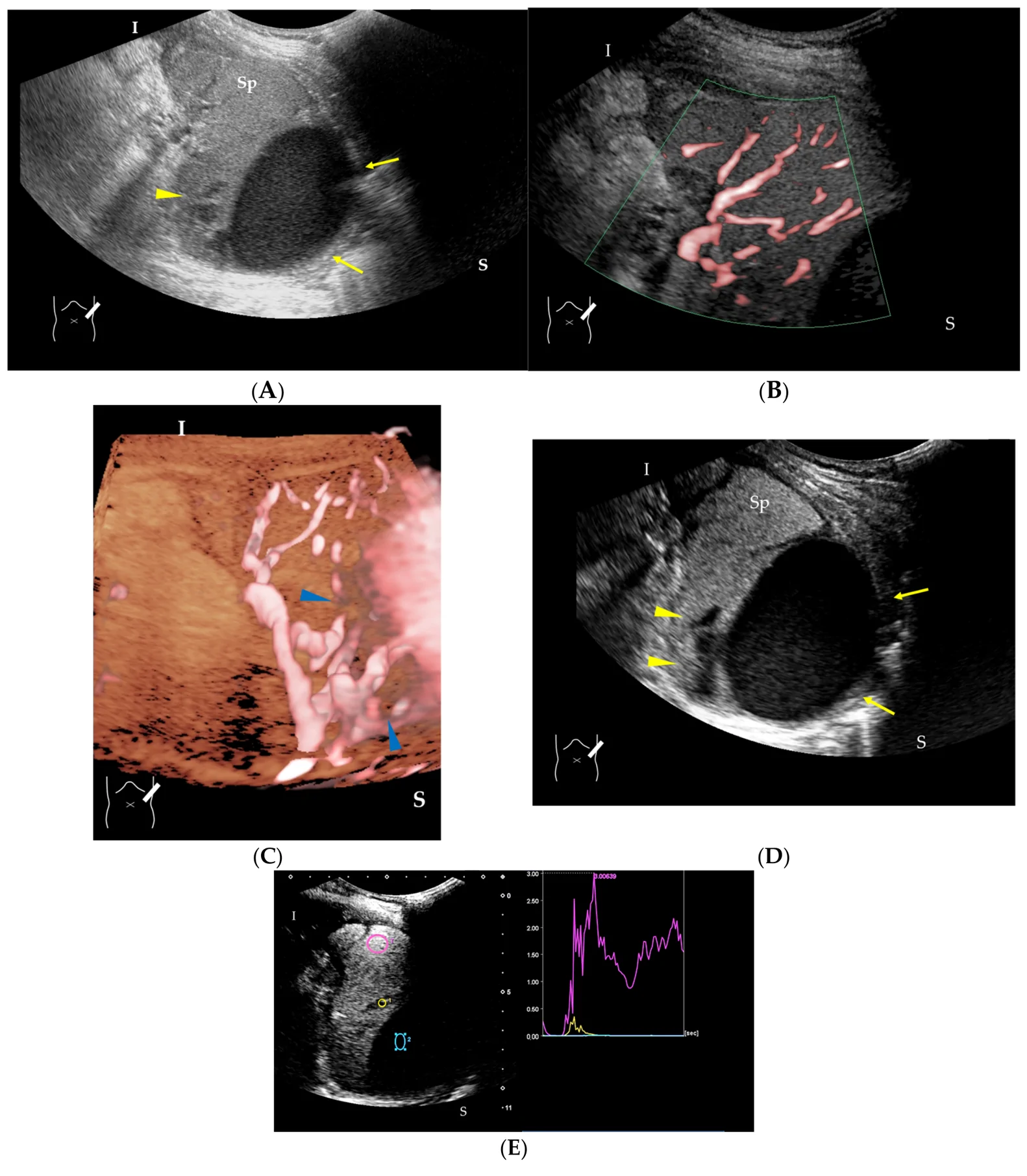
A case of posttraumatic splenic abscess. (**A**) B-mode ultrasonography demonstrates a splenic abscess (yellow arrows) and surrounding damaged parenchyma (yellow arrowheads) (Sp, spleen). (**B**) MVFI shows fine splenic blood flow in the normal parenchyma. (**C**) Three-dimensional MVFI reveals narrowing and interruption of blood flow within the damaged splenic parenchyma (blue arrowheads). (**D**,**E**) On CEUS, the splenic abscess appears as a non-enhancing area (yellow arrows), and the damaged parenchyma is clearly delineated (yellow arrowheads). These findings are also demonstrated on the time–intensity curve (TIC) analysis (red circles, normal splenic parenchyma; yellow circles, damaged parenchyma; blue circles, splenic abscess). In this case, the combination of B-mode US, MVFI, and CEUS was effective in comprehensively assessing the disease characteristics. I, inferior; S, superior.

**Figure 15:**
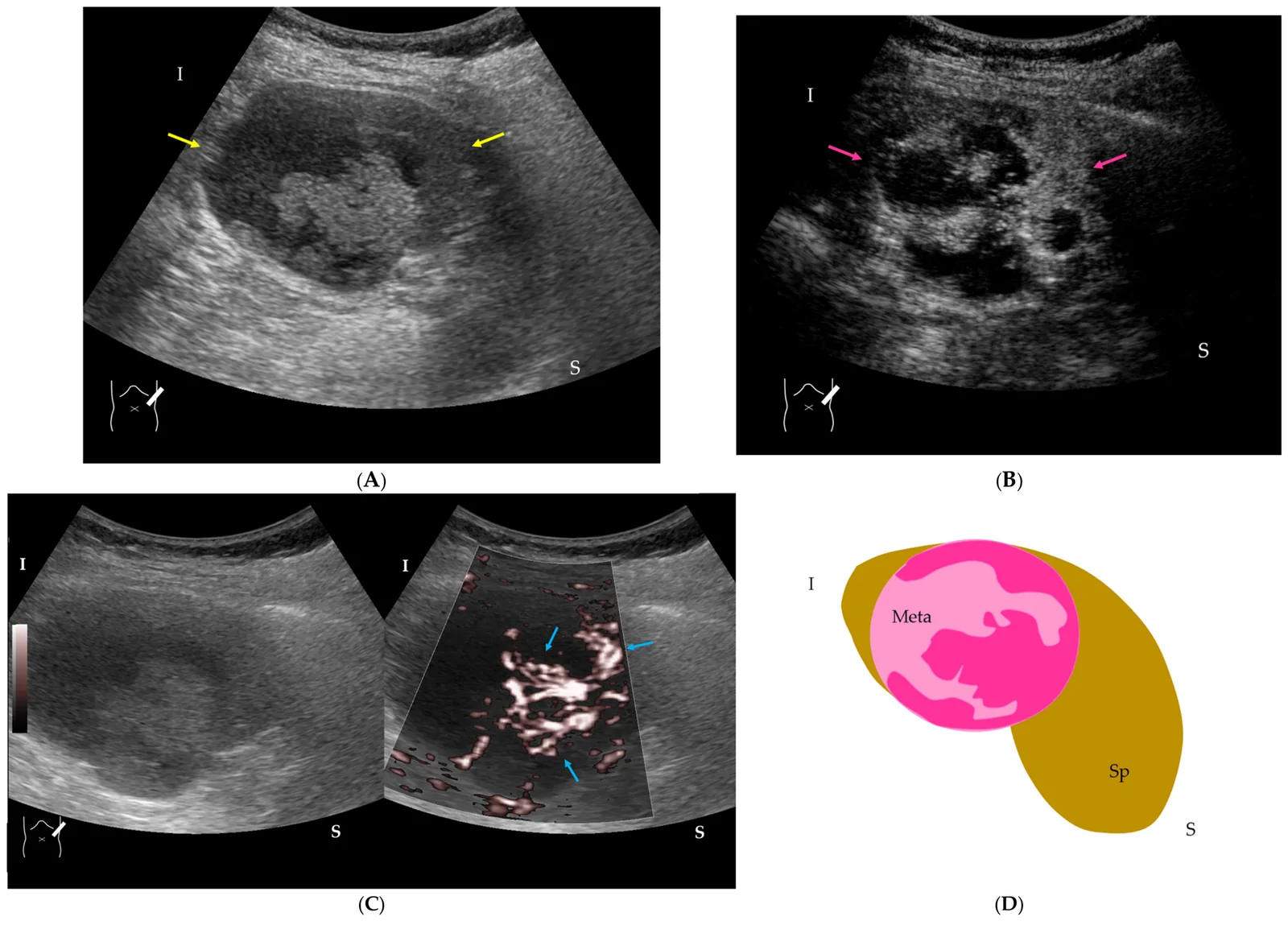
Representative case of successfully surgically resected splenic metastasis from ovarian cancer. (**A**) B-mode US demonstrates a tumor (yellow arrows) in the inferior pole of the spleen with a tendency toward internal necrosis. (**B**) On CEUS, the lesion shows heterogeneous enhancement with extensive non-enhancing areas, suggesting splenic metastasis with marked intratumoral necrosis (pink arrows). (**C**) MVFI performed after CEUS demonstrated abundant tumor vascularity within the solid component of the tumor (blue arrows). (**D**) Schematic illustration of this case (Meta, metastasis; Sp, spleen). In this case, massive intratumoral necrosis was indicated by the combined use of B-mode US and CEUS. I, inferior; S, superior.

The following representative cases are illustrative institutional cases retrospectively selected from routine clinical practice. They are presented for educational purposes and were not used to assess diagnostic performance (Figure 10, Figure 11, Figure 12, Figure 13, Figure 14 and Figure 15). These selected cases are illustrative examples and should not be interpreted as evidence of diagnostic accuracy or generalizability.

## 7. Discussion

The future of splenic ultrasonography lies not in a single imaging technique but in a multiparametric approach that integrates morphological, vascular, perfusion, and mechanical information.

The spleen is an intraperitoneal organ located in the left hypochondrium between the gastric fundus and the left hemidiaphragm. Its functions include hematopoiesis, removal of defective blood cells, and protection against infection [6]. Although requests for US examination of the spleen are relatively uncommon, US practitioners must be familiar with its normal appearance and abnormal US findings in the context of a wide range of diseases [3,4,5,6,36,37,38,39,41,42,43,44,45,46,47], as splenic US can yield many important insights that can improve patient care.

The spleen itself has a markedly irregular shape, macroscopically described as resembling the “head of a jellyfish” [6,48,49,50]. The total splenic volume has been reported to be approximately 150–200 mL; however, it is well recognized that the splenic volume can change in response to various physiological stresses, such as food intake and physical exercise [6,48,49,50].

Accordingly, numerous studies have used ultrasonography to evaluate splenic responses to stress, particularly exercise-induced changes in splenic size [51]. Thus, a stable and reproducible ultrasonographic method for measuring splenic volume is essential to ensure reliable assessment in such settings. The existence of multiple formulas can be attributed primarily to the difficulty of visualizing the entire spleen using conventional ultrasonography [5,10]; in other words, if the whole spleen could be consistently encompassed within a single field of view, some of these limitations could be reduced. From this perspective, the advent of wide-scanning technology is of considerable significance, and it is expected that an increasing number of studies will report splenic volumetry using three-dimensional wide-scanning-based techniques in the future.

Doppler ultrasonography allows quantitative assessment of splenic hemodynamics, including arterial impedance indices and splenic venous flow characteristics. However, these parameters may vary according to physiological and pathological conditions, measurement methodology, and equipment settings. In addition, accurate quantitative assessment may be affected by the tortuous course of splenic vessels, difficulties in maintaining an optimal insonation angle, and variability in vessel diameter and sampling site [48,49,50]. Therefore, quantitative Doppler parameters should be interpreted cautiously rather than regarded as universal reference values.

In contrast, qualitative Doppler assessment based on color-flow imaging and spectral waveform analysis retains substantial clinical value. The splenic artery typically exhibits a pulsatile waveform, whereas the splenic vein shows relatively continuous flow. Importantly, Doppler ultrasonography can provide characteristic hemodynamic information that cannot be obtained from B-mode imaging alone. For example, turbulent or bidirectional flow within a splenic artery aneurysm or pseudoaneurysm can be demonstrated by color Doppler, while spectral Doppler may reveal characteristic abnormal pulsatile or to-and-fro flow patterns. Doppler ultrasonography is also useful for identifying absent or altered flow associated with splenic vein thrombosis [52,53,54,55].

Portable and hand-held ultrasound systems have become increasingly available in recent years, providing rapid bedside imaging with improved diagnostic capability [7]. Their portability is particularly advantageous in emergency and trauma settings, where splenic injury may require immediate assessment. More recently, wireless or cordless ultrasound systems have further expanded the flexibility of bedside imaging by allowing ultrasound data to be transmitted without restricting the operator to the use of conventional cable connections. Although these technological developments are not specific to splenic imaging, their potential value is particularly relevant in emergency assessment of splenic trauma, where rapid bedside evaluation and efficient transmission of imaging information may facilitate subsequent diagnostic decision-making.

In contrast to hepatic elastography, in which increased stiffness is strongly influenced by tissue fibrosis, splenic stiffness is substantially affected by hemodynamic alterations, particularly congestion associated with portal hypertension. Because the spleen is closely connected to the portal venous circulation, increased portal pressure and splenic congestion can result in increased spleen stiffness. Accordingly, spleen stiffness measurement (SSM) has been investigated as a non-invasive marker of portal hypertension and its complications [56,57]. However, spleen stiffness is multifactorial and may also be influenced by tissue composition and fibrosis, inflammatory or infiltrative processes, hematologic disorders, and technical or measurement-related factors.

SSM values are technique-dependent and should not be interpreted using a single universal threshold. Measurements obtained by vibration-controlled transient elastography (VCTE), point shear-wave elastography (pSWE), and two-dimensional shear-wave elastography (2D-SWE) are not directly interchangeable. Even within VCTE, measurements obtained using conventional 50 Hz and spleen-dedicated 100 Hz vibration differ in methodology and validation. Therefore, proposed cut-off values should always be interpreted according to the measurement technique, disease population, and clinical setting in which they were derived, and should not be regarded as universally applicable thresholds [16,18,21,58].

In cirrhosis-related portal hypertension, SSM has been shown to correlate with clinically significant portal hypertension (CSPH) and the presence of gastroesophageal varices. The Baveno VII consensus incorporates SSM into non-invasive assessment of portal hypertension; however, specific thresholds should be applied only within the techniques and populations in which they have been validated. Recent studies using spleen-dedicated VCTE have further supported the clinical utility of SSM for identifying patients at increased risk of CSPH and gastroesophageal varices [22,56,57].

Reported spleen stiffness thresholds are highly technique-dependent. For spleen-dedicated VCTE, particularly using the 100 Hz module, values below approximately 21 kPa and above approximately 50 kPa have been proposed as rule-out and rule-in thresholds, respectively, for clinically significant portal hypertension. However, these thresholds are technique- and population-specific and have been derived predominantly from studies using VCTE in patients with cirrhosis, with substantial evidence obtained in populations with hepatitis B virus-related cirrhosis [21]. Therefore, these thresholds should not be universally applied across different elastography techniques, disease etiologies, or patient populations. Even for cirrhosis, for which spleen stiffness has been most extensively investigated and relatively consistent results have been reported, evidence across the full spectrum of etiologies remains insufficient. Further VCTE studies involving patients with cirrhosis of diverse etiologies are therefore warranted. Compared with VCTE, fewer studies have evaluated spleen stiffness using pSWE or 2D-SWE in patients with cirrhosis, and the reported values and proposed thresholds have been less consistent. Further accumulation of data using pSWE and 2D-SWE is needed before robust and broadly applicable thresholds can be established.

Beyond assessment of the severity of portal hypertension, the relationship between liver and spleen stiffness may provide additional diagnostic information. In non-cirrhotic portal hypertension, a discordant liver–spleen stiffness pattern, characterized by normal or only mildly increased liver stiffness with elevated spleen stiffness, has been reported and may provide a useful diagnostic clue [21,24,32]. In idiopathic portal hypertension (IPH), this discordance has been more directly demonstrated. Furuichi et al., using ARFI-based elastography, showed that the relationship between liver and spleen stiffness may help distinguish IPH from cirrhosis. They used ARFI-based elastography and demonstrated that the spleen-to-liver stiffness ratio was useful for differentiating IPH from other chronic liver diseases. Using a cutoff value of 1.71, the spleen-to-liver stiffness ratio yielded an AUROC of 0.933, with a sensitivity of 94.1%, specificity of 80.0%, and accuracy of 83.9% for differentiating IPH from patients with liver cirrhosis or chronic hepatitis [24]. More recently, Zhou et al., using 2D-SWE, demonstrated that spleen stiffness showed high diagnostic performance for predicting high-risk varices in patients with IPH. At a cutoff value of 44.0 kPa, the AUC was 0.98, with a sensitivity of 93%, specificity of 96%, PPV of 93%, and NPV of 96%. In HBV-infected patients, the corresponding cutoff value was lower (35.2 kPa), with an AUC of 0.96, sensitivity of 100%, specificity of 82%, PPV of 82%, and NPV of 98%. Notably, among patients with high-risk varices, those with IPH had significantly higher spleen stiffness than HBV-infected patients (50.2 vs. 42.9 kPa), whereas liver stiffness was significantly lower in IPH (11.1 vs. 18.3 kPa; both *p* < 0.0001) [21]. These findings further support the potential diagnostic value of a discordant liver–spleen stiffness pattern in IPH.

In contrast, evidence specifically addressing the diagnostic utility of spleen stiffness in left-sided portal hypertension (LSPH) remains limited. Because LSPH is characterized by localized portal hypertension, usually associated with splenic venous obstruction, with relatively preserved hepatic parenchyma, a discordant pattern of relatively normal liver stiffness and elevated spleen stiffness may be clinically plausible. However, this concept has not been sufficiently validated in LSPH-specific studies, and its clinical role in this setting remains to be established [59,60]. This should therefore be regarded as a suggestive imaging pattern that warrants further evaluation rather than as a diagnostic criterion for LSPH. Splenic elastography may provide complementary information when interpreted together with splenomegaly, splenic venous abnormalities, collateral circulation, and other clinical and imaging findings. Further studies are required to establish its diagnostic value in LSPH.

Additional approaches combining spleen stiffness with other parameters, including platelet count, have also been proposed to improve diagnostic performance. Nevertheless, from a practical standpoint, SSM may be particularly useful for two complementary purposes: assessment of the severity of portal hypertension and evaluation of the relationship between liver and spleen stiffness. These measurements should be interpreted together with clinical findings, laboratory data, and conventional ultrasonographic evidence of portal hypertension rather than as stand-alone diagnostic criteria.

Finally, elevated spleen stiffness is not specific to portal hypertension. Markedly increased values have also been reported in hematologic disorders. Sansone et al. reported significantly higher spleen stiffness in patients with myelofibrosis (MF) than in healthy volunteers using both pSWE (40.9 vs. 26.3 kPa) and 2D-SWE (34.9 vs. 20.1 kPa; both *p* < 0.001). These findings suggest that spleen stiffness may also reflect hematologic and infiltrative changes and may contribute to disease assessment and evaluation of treatment response. Therefore, hematologic disease should also be considered when unexpectedly high spleen stiffness values are encountered [61,62].

Doppler ultrasonography has become an indispensable tool for assessing portal venous hemodynamics. However, conventional Doppler techniques have inherent limitations—particularly their relatively low sensitivity for detecting slow-flow vessels and limited spatial resolution—which make detailed portal and splenic microcirculation evaluations difficult. To overcome these limitations, microvascular flow imaging (MVFI) was recently introduced, encompassing several diagnostic technologies that mainly utilize two different techniques: B-flow imaging and Superb Microvascular Imaging (SMI). As these methods are based on fundamentally different principles, understanding their respective mechanisms is important for appropriate image interpretation. The principles of B-flow imaging and SMI are briefly described below.

The core principle of SMI is spatio-temporal correlation processing (Figure 4). MVFI can demonstrate altered, reduced, or low-velocity flow in situations where conventional Doppler assessment is inconclusive; however, these findings do not by themselves establish the presence or cause of thrombosis and should be interpreted together with B-mode, conventional Doppler, and clinical findings, as well as other imaging findings, when appropriate [13,14]. MVFI may improve visualization of vascular architecture in some hypovascular lesions.

MVFI enables visualization of low-velocity microvascular flow that is difficult to detect with conventional color Doppler imaging. SMI is optimized for low-velocity flow imaging, with the velocity scale typically set below approximately 2.5 cm/s [13]. Despite its advantages, quantitative diagnostic performance data for splenic applications of MVFI, including sensitivity, specificity, PPV, and NPV, remain limited. Therefore, its diagnostic performance should not be considered equivalent to that of CEUS, and further prospective studies are required. Thus, MVFI and CEUS should be regarded as complementary rather than interchangeable techniques for the assessment of splenic vascularity.

The greatest advantage of MVFI is its ability to display images at an ultra-high frame rate (approximately 50–80 frames/s), enabling the real-time evaluation of dynamic flow changes. This may improve the ability to characterize minimally altered blood flow states and the internal vasculature of tumors. It should be noted that MVFI offers certain advantages that CEUS does not. In CEUS examinations, the mechanical index (MI) of the reference image is intentionally kept low, resulting in less distinct B-mode visualization within the field of view. In contrast, MVFI allows for prolonged and stable observation of selected regions from multiple scanning angles under consistent imaging conditions. This is particularly useful when CEUS is performed subsequently, as MVFI can help to determine the optimal imaging plane for contrast-enhanced observation.

CEUS is also widely used in routine clinical practice, particularly for differential diagnosis of hepatic tumors [29]. In contrast to hepatic CEUS, for which standardized diagnostic frameworks are available, splenic CEUS currently lacks a widely accepted standardized classification system. Therefore, interpretation of splenic lesions should integrate baseline B-mode appearance, lesion morphology, arterial enhancement pattern and homogeneity, the timing and degree of washout, and the clinical context, based on the available spleen-specific evidence [32,33,34].

Regarding the temporal phases of splenic CEUS, unlike hepatic CEUS—which is generally divided into the arterial phase, portal venous phase, and late vascular phase—the splenic enhancement pattern is usually divided into the arterial phase, occurring approximately 10–35 s after contrast injection, followed by the parenchymal phase.

The differences between splenic and hepatic enhancement patterns are thought to be mainly attributable to differences in tissue composition (i.e., the liver is composed primarily of regularly arranged hepatocytes, while the spleen consists of red pulp and white pulp), in addition to differences in vascular architecture; in particular, the liver possesses two inflow vessels (the hepatic artery and portal vein), whereas the spleen has only one inflow vessel (the splenic artery).

In general, during the arterial phase, the splenic parenchyma exhibits heterogeneous enhancement known as the “zebra sign”; in light of this, observation during the period at approximately 3–5 min after contrast injection is considered most suitable for lesion evaluation [33,34].

Key clinical roles of splenic CEUS include characterization of splenic inhomogeneity, confirming splenic infarction, confirming accessory spleen or splenosis, detection and characterization of focal lesions, and evaluation of the spleen in oncologic or post-trauma patients [32,33,34,35,36,37,38,39].

CEUS enhancement patterns of splenic lesions show considerable overlap between benign and malignant entities; therefore, these findings should not be regarded as definitive diagnostic criteria. Nevertheless, as a general tendency, malignant splenic lesions more often demonstrate hypo- or isoenhancement during the arterial phase, followed by relatively early washout during the parenchymal phase, whereas benign lesions more commonly show hyperenhancement with persistent enhancement or absent, mild, or delayed washout. These findings should always be interpreted in conjunction with B-mode appearance, other imaging findings, and the clinical context [32,33,34,37,40,42].

Regarding the differentiation of focal splenic lesions, Yang et al. reported the largest series to date, comprising 123 lesions (83 benign and 40 malignant lesions). Arterial-phase hyperenhancement was observed substantially more frequently in benign lesions than in malignant lesions (38.6% vs. 2.5%), and parenchymal-phase hyperenhancement also differed between benign and malignant lesions (15.7% vs. 0%, respectively). Additional features included regular-shaped enhancement (59.6% vs. 27.5%) and wedge-shaped enhancement (15.7% vs. 0%). When these features were combined as predictors of malignant splenic lesions, the sensitivity, specificity, positive predictive value (PPV), negative predictive value (NPV), and accuracy were 55.0% (22/40), 100.0% (83/83), 100.0% (22/22), 82.2% (83/101), and 85.4% (105/123), respectively [63]. However, differences in CEUS findings among individual types of malignant splenic lesions were not evaluated in detail. Furthermore, terms such as “rapid enhancement” and “complete enhancement” were used without clearly standardized definitions. Thus, although CEUS shows potential for differentiating benign from malignant splenic lesions, several aspects of lesion-specific enhancement remain insufficiently characterized, and further studies using standardized definitions and detailed analyses are required to improve the qualitative characterization of focal splenic lesions.

Differences in tissue composition, vascular architecture, and the presence or absence of sinusoidal reticuloendothelial components may contribute to differences in CEUS enhancement patterns.

One of the most important clinical applications of splenic CEUS is the diagnosis of splenic infarction. At the onset of infarction, lesions are often difficult to detect on conventional B-mode ultrasonography. In contrast, CEUS typically demonstrates a well-defined wedge-shaped non-enhancing area extending to the splenic margin, making the diagnosis relatively straightforward [32,33,34]. Several small studies comparing CEUS with CT and/or MRI have reported its favorable diagnostic performance for focal splenic lesions. In one retrospective single-center study, CEUS showed 100% sensitivity, specificity, positive predictive value, and negative predictive value for splenic infarction compared with CT. However, direct comparative evidence among CEUS, CT, and MRI remains limited, and larger multicenter studies are required to clarify their relative and complementary diagnostic roles [64,65,66].

CEUS is also valuable for the diagnosis of accessory splenic tissue. As accessory spleens exhibit enhancement patterns identical to those of the native spleen during all enhancement phases, this characteristic finding serves as an important diagnostic clue and can facilitate confident identification of ectopic splenic tissue [32,33,34].

Elucidating these mechanisms will require the accumulation of many cases with careful correlation between CEUS findings and histopathological features. At present, although several larger observational studies are available, the overall spleen-specific CEUS literature remains limited and includes many case reports and small case series. Therefore, further multicenter studies with larger patient populations and standardized CEUS criteria are needed to establish diagnostic performance and enable reliable comparison with other imaging modalities.

In addition to the technique-specific limitations discussed above, ultrasonography has several inherent limitations that should be considered when evaluating the spleen. Image quality and visualization may be impaired by obesity, overlying bowel gas, the deep location of the spleen, and interference from the ribs. In addition, ultrasonography is operator-dependent, and image acquisition and interpretation may vary according to the examiner’s experience and technical expertise. These factors may affect the reproducibility and diagnostic performance of splenic ultrasonography (Table 2).

**Table 2:** Summary of current ultrasonographic techniques for splenic assessment.

Technique	Clinical Application	Current Evidence	Strength	Major Limitations
WS	Whole spleen visualization, size assessment	Limited	Wide FOV	Limited evidence, system dependence
Doppler	Hemodynamics, thrombosis	Established clinical experience	Real-time flow, measurement	Insufficient detection of slow flow
MVFI	Detection of slow flow/microvascular visualization	Limited	High sensitivity to slow flow	Small ROI, depth dependance
Elastography	Portal hypertension	Moderate and growing	Quantitative	Technique specific threshold
Hand-held US	Bedside screening, POCUS	Established mainly emerging, bedside, education	Portability	Degraded image quality
CEUS	Perfusion, infarction, focal lesions	Limited	Real-time perfusion	Extension of time

WS, wide scanning; FOV, field of view; MVFI, microvascular flow imaging; ROI, region of interest; POCUS, point of care ultrasound; CEUS, contrast-enhanced ultrasound.

## 8. Conclusions

Recent advances in ultrasonography, including WS, MVFI, elastography, and CEUS, have expanded the possibilities for splenic assessment beyond conventional B-mode and Doppler imaging. WS may facilitate more comprehensive visualization of the spleen and provide a useful platform for subsequent Doppler, MVFI, and CEUS assessment, while elastography and CEUS provide complementary information on portal hemodynamics and focal splenic lesions, respectively. However, spleen-specific evidence for several of these techniques remains limited, and their integrated use should currently be regarded as a proposed practical approach rather than an established diagnostic framework. Further prospective comparative studies are needed to define their optimal roles and validate their combined clinical application.

## Figures and Tables

**Table 1:** Comparison of representative microvascular imaging techniques and CEUS in splenic ultrasonography.

Technique	Definition/ Principle	ROI Requirement	Penetration	Repeatability	Quantitative Capability	Vendor	Primary Clinical Applications
MVFI	Generic term of microvascular imaging	Small ROI	Limited	Excellent	Limited	Multiple vendors	Microvascular imaging
SMI	Clutter suppression	Small ROI	Limited	Excellent	Limited	Canon	Tumor vascularity, inflammation
B-flow	Coded excitation	Small ROI	Limited	Excellent	Limited	GE HealthCare	Vascular morphology
CEUS	Contrast-enhanced perfusion imaging	Whole imaging field	Good *	Limited	Excellent (TIC)	Multiple vendors	Lesion characterization, perfusion assessment

* Although CEUS is also affected by attenuation in deep lesions, penetration can be improved by optimizing imaging parameters (e.g., lower transmit frequency and contrast-specific settings). ROI, region of interest; MVFI, microvascular flow imaging; SMI, superb micro-vascular imaging; CEUS, contrast-enhanced ultrasound; TIC, time intensity curve.

## Data Availability

No new data were created or analyzed in this study. Data sharing is not applicable to this article.

## Abbreviations

The following abbreviations are used in this manuscript:

CEUS	contrast-enhanced ultrasound
CT	computed tomography
FOV	field of view
IPH	idiopathic portal hypertension
LI-RADS	Liver Imaging Reporting and Data System
MF	myelofibrosis
MI	mechanical index
MRI	magnetic resonance imaging
MVFI	Microvascular flow imaging
NPV	negative predictive value
POCUS	point of care ultrasound
PPV	positive predictive value
pSWE	point shea-wave elastography
ROI	region of interest
SWE	shear-wave elastography
TIC	time–intensity curve
2D-SWE	two-dimensional shear-wave elastography
US	ultrasound
VCTE	vibration-controlled transient elastography
WS	wide scanning
